# Causes of Death of Adults and Elderly and Healthcare-seeking before Death in Rural Bangladesh

**DOI:** 10.3329/jhpn.v28i5.6161

**Published:** 2010-10

**Authors:** Nurul Alam, Hafizur Rahman Chowdhury, Monirul Alam Bhuiyan, Peter Kim Streatfield

**Affiliations:** ^1^ Health and Demographic Surveillance Unit; ^2^ Matlab Hospital; ^3^ Population Programme, ICDDR,B, GPO Box 128, Dhaka 1000, Bangladesh

**Keywords:** Adult, Causes of death, Elderly, Healthcare-seeking behaviour, Mortality, Rural health, Bangladesh

## Abstract

The health system of a country needs to be adjusted to patterns of morbidity and mortality to mitigate the income-erosion consequences of prolonged ill-health and premature death of adults. Population-based data on mortality by cause are a key to modifying the health system. However, these data are scarce, particularly for rural populations in developing countries. The objectives of this study were to determine the burdens of health due to major causes of death obtained from verbal autopsy of adults and the elderly and their healthcare-seeking patterns before death in a well-defined rural population. There were 2,397 deaths—613 were among adults aged 15-59 years and 1,784 among the elderly aged 60^+^ years—during 2003-2004 in the health and demographic surveillance area in Matlab, a rural area of Bangladesh. Trained interviewers interviewed close relatives of the deceased using a structured verbal-autopsy questionnaire to record signs and symptoms of diseases/conditions that led to death and medical consultations before death. Two physicians independently assigned the underlying causes of deaths with disagreements resolved by a third physician. The physicians were able to assign a specific cause in 91% of the cases. Rates and proportions were used for estimating the burden of diseases by cause. Of all deaths of adults and the elderly, communicable diseases accounted for 18% and non-communicable diseases for 66%, with the proportion of non-communicable diseases increasing with age. Leading non-communicable diseases were diseases of the circulatory system (35%), neoplasms (11%), diseases of the respiratory system (10%), diseases of the digestive system (6%), and endocrine and metabolic disorders (6%), all of which accounted for 68% of deaths. Injury and other external causes accounted for another 5% of the deaths. During terminal illness, 31% of the adults and 25% of the elderly sought treatment from medical doctors, and 14% of the adults and 4% of the elderly died in healthcare facilities. The findings suggest that the health managers and policy-makers of Bangladesh should recognize the importance of prevention and management of chronic diseases and place it on the health agenda for rural people.

## INTRODUCTION

In 2004, an estimated 58.8 million deaths occurred globally, of which 27.7 million were among females and 31.1 million among males (1). Of every 10 deaths, six were due to non-communicable conditions; three due to communicable, reproductive, or nutrition-related conditions; and one due to injuries. In countries with developing and transitional economies, non-communicable diseases (NCDs), along with a few emerging and re-emerging diseases, such as malaria and tuberculosis, show increasing trends, and other infectious diseases show decreasing trends (2,3). Results of studies on mortality in South Asian countries indicate a transition in patterns of mortality with increase in share of NCDs. Studies on mortality in rural India and Bangladesh also revealed an increased prominence of NCDs (4,5).

An increase in the proportion of NCDs and injuries in recent years has drawn the attention of international organizations. The World Health Organization (WHO) (2008) projected that NCDs would account for at least seven of 10 deaths occurring in developing regions by 2020. The WHO also stated that injuries—both unintentional and intentional—would become more prevalent and were projected to show rates equal to those of mortality due to infectious diseases by 2020 (6).

In developing countries, NCDs not only tend to increase but also appear earlier in life (7). Chronic NCDs, including diabetes and hypertension, and chronic obstructive pulmonary disorder (COPD) require continuous care. Care costs money and time. Unmet needs for medical care are critically higher for chronic illnesses than for acute illnesses in rural Bangladesh (8). Both prolonged ill-health and premature death of main income-earners of households lead to erosion of income with consequences at both micro- and macro-levels (9). Death of young adults is shown to have deepened the spiral of household poverty in rural Bangladesh (10). Thus, control of NCDs has an important role to alleviate poverty.

Planning logistics for controlling NCDs requires up-to-date and reliable population-based statistics of morbidity and mortality by cause. Such statistics, to our knowledge, are lacking, particularly for adolescents, adults, and the elderly in Bangladesh. Most (90%) deaths in rural areas occur in the home and have no death certificate from which one can derive cause of death (11). The national sample vital registration system records cause of death (COD) reported by family members of the deceased (12). Reliance on lay reports of COD, however, coupled with a high proportion of unspecified cause (such as old age), limits the use of national data on COD for planning health services and logistics.

In developing countries where registration of deaths is incomplete and real autopsies are not feasible, verbal autopsy (VA) is a surrogate for population-based death certificates to derive COD. Studies in India, China, and South Africa validated VA for the assessment of COD for adults and the elderly and concluded that VA-derived COD is reliable with respect to the broader cause categories (13–15). VA is increasingly used for deriving COD for setting priorities, planning health services, and monitoring and evaluating the performance of the health system (16). In Bangladesh, where registration of deaths is incomplete and death certificate does not exist, VA is a practical low-cost option to generate population-based death certificate and COD. To generate a database of COD and healthcare responses to fatal illnesses in a well-defined rural population, the International Centre for Diarrhoeal Disease Research, Bangladesh (ICDDR,B) introduced a standard VA in 2003 in Matlab, a rural area of Bangladesh. A rigorous and dynamic health and demographic surveillance system (HDSS) has been operating in Matlab since 1966. The objectives of the present study were to estimate the health burdens of adults and the elderly due to broad disease categories and to assess the healthcare-seeking patterns before death in rural Bangladesh using VA data.

## MATERIALS AND METHODS

Data for the study were drawn from Matlab where ICDDR,B has been maintaining the HDSS since 1966. Matlab is 55 km southwest of Dhaka—the capital city of Bangladesh. The HDSS covered a population of 224,762 in 142 villages in 2005 (17). The majority of the population are Muslims (88%), and 12% are Hindus. Agriculture, aquaculture, and trade are the main occupations of men. Women usually do household chores, including cooking, cleaning, and caring for children. Despite the low level of economic development, Matlab has made good progress in lowering the mortality rate in children aged less than five years (50 per 1,000 livebirths in 2007 compared to 200 per 1,000 livebirths in 1978) and raising life expectancy at birth—67 years for males and 69 years for females in 2007 compared to 56 and 54 years respectively in 1978 (18).

The female community health research workers of the HDSS detect and record vital events, including death, through monthly (bi-monthly since 2008) visits to households. The HDSS introduced three structured VA questionnaires for collecting information on neonates, children, adolescents, and adults. These questionnaires were developed by the WHO and modified by INDEPTH for its member surveillance sites in 2003. The VA tools in English were customized and translated into Bangla for use in the field. A public-health physician and a medical demographer trained six field research assistants (3 males and 3 females) of non-medical background and a medical assistant (with 3 years of training in medicine in the public sector) on modular VA tools, followed by two days of field practice. Trained field research assistants interviewed the closest caretakers of the deceased using the VA questionnaire within 6-12 weeks after the date of death. The medical assistant regularly supervised the fieldwork of the field research assistants. The medical assistant and the public-health physician were available to provide technical support, such as clarification of questions when required. The VA respondents were caretakers/relatives who had lived with the deceased in the same household around terminal illness or death.

### VA review and assessment of COD

The HDSS recorded 3,129 deaths during 2003-2004, and VA was obtained for 3,125 (99.9%) deaths. Analysis of COD included 2,396 of 2,397 deaths of adults. In 2005, a physician reviewed each VA sheet and filled out the death certificates from the list of three character categories from the International Classification of Diseases (version 10) (ICD-10) codes, also recording notes of the points in favour of their diagnoses. The death certificate has four rows: disease or condition directly leading to death (or direct cause); morbidity conditions, if any, giving rise to the direct cause (or the underlying cause); and other significant conditions contributing to death but not related to the disease or condition causing it (or associated cause). To assess consistency in determining COD, two physicians with some experience in the line reviewed VA sheets of non-injury-related deaths in 2007 (Fig.). One reviewed the VA sheets from 2003, and another reviewed the VA sheets from 2004 independently—both serving as second physician-reviewers. The underlying COD determined by the first and the second physician-reviewer matched for 59.3% of the 2,396 deaths. The third physician-reviewer blindly reviewed the VA sheets with the mismatched underlying COD (n=975). The mismatched VA sheets from 2003 were reviewed by one of the second reviewers who had not reviewed the VA sheets from 2003 as a second physician-reviewer before, and the mismatched VA sheets from 2004 were reviewed by the other second reviewer who had not reviewed the VA sheets from 2004 as a second physician-reviewer before. The underlying COD of any two of the three physicians matched for 628 (64.4%) cases and did not match for 347 cases. The last two physicians jointly reviewed these mismatched VA sheets as final reviewers, and their assessment was treated as final.

**Fig.**
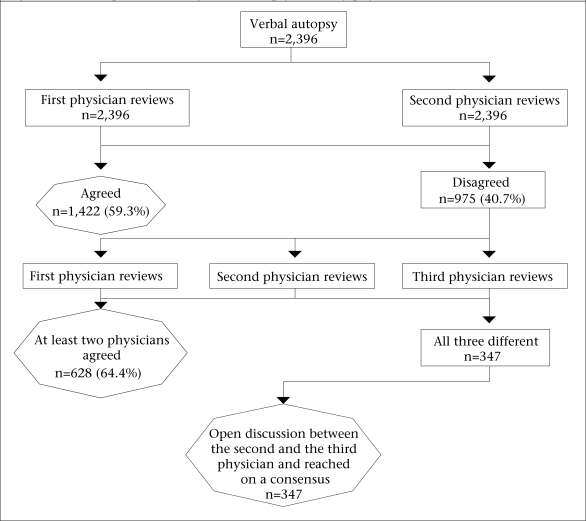
Schematic steps in reviewing verbal autopsy sheets by physician

### Analysis of data

Proportion and rate (per 1,000 person-years) of deaths were used for showing the age and sex patterns in the risk of dying. Analysis of COD included the main underlying COD and healthcare-seeking during fatal illnesses for deaths of adults and the elderly only. The underlying CODs were grouped into communicable diseases, non-communicable diseases, pregnancy-related deaths, injury, or unspecified category. They were attributed to the main underlying causes defined by the chapter headings in ICD-10. If more than 50 deaths fell within one group, a further breakdown of the main components is provided. The percent distribution of CODs by age and sex indicated the age and sex differentials in cause-specific mortality.

## RESULTS

Of the 3,129 deaths occurred in the HDSS area during 2003-2004, 16% were infants, 7.3% were children aged 1-14 years, 19.6% were adults aged 15-59 years, and 57% were elderly aged 60+ years (Table 1). The death rates were higher among males than among females, with a larger difference at ages of 15-59 years (when mortality of males was 1.45 times higher than mortality of females) than at ages of 60+ years (when mortality of males was 1.23 times higher than mortality of females). The risk of mortality was also highly age-dependent; the rate for the elderly people was 18 times higher than the rate for adults.

**Table 1.** Distribution of deaths and death rates by age and sex in 2003-2004

Age-group (years)	No. of deaths		% of all deaths		Rates/1,000 person-years	
	Male	Female	Male	Female	Male	Female
<1	263	239	15.7	16.4	46.7	43.8
1-4	78	71	4.7	4.9	3.5	3.4
5-14	34	47	2.0	3.2	0.7	0.9
15-59	345	268	20.7	18.4	2.9	2.0
60+	950	834	56.9	57.2	52.3	42.6
All	1,670	1,459	100	100	7.8	6.2

The CODs were analyzed for adult and elderly age-groups only. The VA physicians assigned a specific underlying cause to 2,168 (90.5%) of the 2,396 deaths, and a non-specific cause to 9.5% (Table 2). A striking feature of the patterns of broader COD was the prominence of non-communicable conditions, which accounted for 66.1% of the deaths compared to communicable conditions, which accounted for 18.3% of the deaths. Injury or external causes accounted for 4.9% of the events of death, and conditions relating to pregnancy accounted for 10.1% of the events of death of adult (aged 15-59 years) females. Males had higher mortality rates than females due entirely to higher mortality from communicable diseases and injury at ages of 15-59 years (p<0.01).

**Table 2.** Distribution of deaths* of adults and the elderly by broader cause category and sex, 2003-2004

Broader COD category	Sex of the deceased			Age 15-59 years		Age 60+ years	
	Total	Male	Female	Male	Female	Male	Female
Communicable diseases	18.3	19.6	16.8	18.6	14.6	20.0	17.5
Non-communicable diseases	66.1	65.6	66.7	63.5	65.3	66.4	67.1
Pregnancy-related	1.1	NA	2.4	NA	10.1	NA	NA
Injury or external cause	4.9	5.5	4.3	11.3	7.5	3.4	3.2
Unknown	9.5	9.3	9.8	6.7	2.6	10.2	12.1
Total							
%	100	100	100	100	100	100	100
No.	2,396	1,294	1,102	345	268	949	834
Chi-square (degree of freedom)		36.4 (4), p<0.01		43.7 (4), p<0.01		3.0 (3), p=0.4	

*Verbal autopsy could not be collected for one elderly death; COD=Cause of death; NA=Not applicable

### Gender and causes of death

The most common COD in this rural population was diseases of the circulatory system (35.1%), which showed a significant gender difference (Table 3). This category primarily included cerebro- vascular diseases (25%) and ischaemic heart diseases (5.1%). The other diseases of the circulatory system accounted for another 5% of the deaths, and these were other forms of heart diseases (3.1%), rheumatic heart disease (0.8%), hypertensive disease (0.7%), and diseases of arteries and veins (0.4%). While ischaemic heart diseases were more frequent among men, cerebrovascular diseases were more frequent among women. The second most common cause of death was infectious and parasitic diseases (13.3%), with tuberculosis accounting for 6.1% of the deaths and diarrhoea accounting for 3.7% of the deaths. Men were more frequent victims of tuberculosis compared to women.

**Table 3.** Percentage of events of death of adults and the elderly by cause and sex

COD (and ICD10 codes)	No. of deaths	%*			
		Male	Female	Total	95% CI
Circulatory system (I00-I99)	840	31.5	39.3	35.1	33.1-37.0
Cerebrovascular disease	598	19.9	30.9	25.0	
Ischaemic heart disease	122	7.0	2.8	5.1	
Other circulatory diseases	120	4.6	5.5	5.0	
Infectious and parasitic diseases (A00-B99)	319	15.5	10.8	13.3	12.0-14.7
Diarrhoeal diseases	88	3.1	4.4	3.7	
Tuberculosis	146	8.5	3.3	6.1	
Other infectious diseases	85	3.9	3.2	3.5	
Neoplasm (C00-D48)	274	13.0	9.6	11.4	10.2-12.7
Digestive system	105	5.6	3.0	4.4	
Respiratory system	38	2.6	0.4	1.6	
Ill-defined unspecified site	131	4.8	6.3	5.5	
Respiratory system (J00-J99)	241	11.1	8.4	10.1	8.9-11.3
Asthma	131	6.4	4.4	5.5	
COPD	47	1.9	2.1	2.0	
Acute respiratory distress syndrome	47	2.2	1.6	2.0	
Other respiratory diseases	16	0.9	0.4	0.6	
Digestive system (K00-K93)	144	6.2	5.8	6.0	5.1-7.0
Liver diseases	82	3.2	3.7	3.4	
Other diseases	62	3.0	2.1	2.6	
Endrocrine, nutritional, and metabolic diseases (E00-E90)	143	5.4	6.6	6.0	5.0-6.9
External cause mortality (S00-Y98)	118	5.5	4.3	4.9	4.1-5.8
Self-harm	16	0.7	0.6	0.7	
Transport	18	1.2	0.2	0.8	
Other injury	84	3.6	3.4	3.6	
Genitourinary system (N00-N99)	36	1.8	1.2	1.5	1.0-2.0
Nervous system (G00-G99)	10	0.3	0.5	0.4	0.2-0.7
Congenital malformation (Q00-Q99)	4	0.2	0.2	0.2	
Pregnancy, childbirth, and the puerperium (O00-O99)	27	NA	2.5	1.1	0.7-1.5
Blood/blood forming organ (D50-D89)	4	0.1	0.3	0.2	
Skin and subcutaneous tissue (L00-L99)	3	0.1	0.2	0.1	
Mental and behavioural disorder (F00-F99)	5	0.1	0.5	0.3	
Diseases of bones and joints (M00-M99)	3	0.1	0.2	0.1	
Not elsewhere classified (R00-R99)	224	9.0	9.7	9.4	8.2-10.5
No. of deaths	2,396	1,294	1,102	NA	

*Totals may not add to 100 precisely due to rounding; CI=Confidence interval; COD=Cause of death; COPD=Chronic obstetric pulmonary diseases; ICD=International classification of diseases; NA=Not applicable

The third leading cause was malignant neoplasm (11.4%). It was not always possible to identify the particular organ-system. Malignancies were more frequent in the digestive system (4.4%) than in the respiratory system (1.6%). The fourth leading cause was diseases of the respiratory system (10.1%), with asthma accounting for 4.4% and COPD for 2% of the deaths. The other leading causes were diseases of the digestive system (6.0%), endrocrine and metabolic disorders (6%), and injury and external causes (4.9%). Deaths due to diabetes and hypertension were not very common but would have contributed substantially to the high proportions of cerebrovascular diseases, ischaemic heart diseases, and renal failure. Globally, 54% of strokes and 47% of ischaemic heart diseases were attributable to hypertension (19). The top five causes accounted for 75.9% of the deaths.

### Age and causes of death

Age distribution of the deaths was heavily skewed—25.6% occurred among the adults and 74.4% occurred among the elderly people (Table 4). Some causes were more frequent in the elderly than in the adult age-group. For example, 37.9% of deaths of the elderly compared to 26.7% of deaths of the adults were attributed to diseases of the circulatory system. Deaths due to neoplasm, diseases of the digestive system, and injury were more frequent among the adults while diseases of the respiratory system and endrocrine and metabolic disorders were more frequent among the elderly.

**Table 4.** Percentage of events of death of adults and the elderly by cause

COD (and ICD10 codes)	No.	%*		
		15-59 years	60 + years	All
Circulatory system (I00-I99)	840	26.8	37.9	35.1
Infectious and parasitic diseases (A00-B99)	319	16.0	12.4	13.3
Neoplasm (C00-D48)	274	17.1	9.5	11.4
Respiratory system (J00-J99)	241	4.4	12.0	10.1
Digestive system (K00-K93)	144	9.8	4.7	6.0
Endrocrine, nutritional, and metabolic diseases (E00-E99)	143	2.1	7.3	6.0
External cause mortality (S00-Y98)	118	9.6	3.3	4.9
Genitourinary system (N00-N99)	36	2.6	1.1	1.5
Nervous system (G00-G99)	10	0.8	0.3	0.4
Congenital malformation (Q00-Q99)	4	0.7	0.0	0.2
Pregnancy, childbirth and the puerperium (O00-O99)	27	4.4	NA	1.1
Blood/blood forming organ (D50-D99)	4	0.0	0.2	0.2
Skin and subcutaneous tissue (L00-L99)	3	0.2	0.1	0.1
Mental and behavioural disorder (F00-F99)	6	0.5	0.2	0.3
Diseases of bones and joints (M00-M99)	3	0.2	0.1	0.1
Not elsewhere classified (R00-R99)	224	4.9	10.9	9.4
No. of cases	2,396	613	1,783	100.0

*Totals may not add precisely due to rounding; COD=Cause of death; ICD=International classification of diseases; NA=Not applicable

### Place of death and treatment-seeking

Place of death differed more between age-groups than between sex-groups (Table 5). The adults were more likely to die in a health facility, on the way to a health facility, or at the site of injury than the elderly people.

**Table 5.** Place (%) of death of adults and the elderly by sex, 2003-2004

Age (years) and sex of the deceased	No. of deaths	Place of death (%)			
		Home	Health facility	On the way	Spot of injury
15-59	613	68.8	13.7	10.3	7.2
60+	1,784	88.2	4.0	7.0	0.8
Male	1,295	81.8	8.1	7.5	2.6
Female	1,102	85.0	4.5	8.3	2.2
All	2,396	83.2	6.5	7.8	2.4

Although 88.4% of the deceased made a visit to a healthcare provider during terminal illness, untrained village practitioners, including drug-sellers, were consulted more often (57.2%) than medical doctors (26.2%) (Table 6). Paramedics, homeopaths, and spiritual healers were seldom consulted. Age and gender influenced the quality of sickness care; consultation with medical doctors was higher for the adults than for the elderly (30.9% vs 24.7%) and higher for the males than for the females (31.7% vs 22.1%).

**Table 6.** Medical consultation (%) during terminal illness by age and sex, 2003-2004

Age and sex of the deceased* (and no. of cases)	None	Doctors	Paramedics	Village doctors†	Homeopath	*Kabiraj* (herbalist)	Spiritual healer
15-59 years (n=569)	11.4	30.9	1.4	49.9	1.8	4.2	0.3
60+ years (n=1,770)	11.7	24.7	1.8	59.1	0.7	1.7	0.2
Male (n=1,261)	10.0	30.3	1.7	54.8	0.9	2.1	0.3
Female (n=1,078)	13.5	21.4	1.8	59.3	1.1	2.7	0.2
All (n=2,339)	11.6	26.2	1.7	56.9	1.0	2.3	0.3

*Excluded 57 deceased who died of accident or injury on the spot;

†Untrained practitioners of allopathic medicine and allopathic drug-sellers

## DISCUSSION

The results of the study provide, for the first time, a comprehensive picture of broader cause categories of deaths of adults and the elderly in a well-defined rural population in Bangladesh. The results contribute to the existing knowledge. However, their use depends on the quality (measured with reliability and validity) of VA-derived COD. The quality of COD depends on the quality of VA interviews and VA coders. The VA interviewers had extensive experience in recording the history of signs and symptoms of diseases that led to death and were familiar with the local terms for signs and symptoms of these diseases. They received extensive training on the VA tools. They conducted VA interviews with close family members of the deceased (spouse 29%, son/daughter/daughter-in-law 53%, parents 5%, and brother/sister 3%), chosen from among family members who were present during the last illness and were able to describe healthcare sought and signs and symptoms leading to death. Exceptions were made for deaths due to violence. The researchers examined the reliability of verbal interviews in terms of work experience, gender, and educational characteristics of VA interviewers at the onset of the study in a small sample. The VA physicians have experience in reviewing VA and assigning ICD-10 codes for the other projects at ICDDR,B. However, the use of physicians in reviewing VA and assigning COD is less likely to be practised in resource-constrained settings.

Validity of the VA-derived COD is usually assessed by comparing with diagnosis in the hospital, which serves as a (gold standard) reference. As we did not have hospital diagnoses, we were not able to assess validity, which can be considered a limitation of the study. Results of a validation study with deaths of a sample of 796 adults in Tanzania, Ethiopia, and Ghana showed that, compared to the gold standard, death certificates, hospital records, and physician-review of verbal autopsies had a high diagnostic accuracy for cardiovascular diseases (cause-specific mortality fraction within 10-19%) and neoplasms (<10%) (20).

Due to a lack of comparable population-based data on COD for adults and the elderly across settings and times, consistency of the pattern of the VA-derived COD could not be determined. Even if available, the differences in methodology, instruments used, and determination of COD hinder comparison across studies. Acknowledging these differences, results of other studies showed a declining trend of CDs and an increasing trend of NCDs in recent decades. The share of deaths due to CDs among adult women in Matlab had declined from 32% in 1976-1985 to 15% in 1996-1997 (21,22) and to 18% in this study from 2003 to 2004. On the other hand, there was an increased prominence of NCDs. In 9.5% of the deaths, the VA physicians could not assign a specific cause, or reported that the cause was ill-defined. In their seminal work on the global burden of disease, Murray and Lopez advocated for the redistribution of deaths ascribed to ‘symptoms, signs and ill-defined conditions’ (23). Redistribution of the undetermined causes in the Matlab sample raises the proportion of deaths due to NCDs from 66% to 76%.

The results of the present study, despite some limitations, showed not only the prominence of NCDs but also variations by age and sex in the health burdens of specific NCDs, tuberculosis, and injuries in rural areas. These findings indicate a need for different priorities and public-health responses. Parallel sex differentials were observed in the distribution of the risk factors of NCDs in rural Bangladesh (24). For example, more men aged 25-64 years used tobacco products (68.2% vs 32.7%) than women of similar ages, who were more often overweight (15.2% vs 10.8%) and more often had hypertension (14% vs 6.4%) or heart problems (10.5% vs 4.9%).

The increasing prominence of NCDs compared to CDs has serious economic consequences for families and for socioeconomic development of the country. The pace of increase of NCDs in the future depends on the pace of demographic transition (a 10-fold increase in the size of the population aged 60 years and older during this century), the control of infectious diseases, and socioeconomic development. If the rates of mortality of adults, which are due largely to NCDs, are to be reduced further, prevention and management of chronic disease conditions, such as cardiovascular diseases, hypertension, diabetes, cancer, COPD, and tuberculosis need further improvement. Most NCDs require expensive specialized facilities, skilled health workers, and sophisticated equipment for their diagnosis and treatment.

Many NCDs are, however, amenable to prevention through behaviour changes. Lifestyle and behaviours are linked to 20-25% of the global burden of diseases, which is likely to increase rapidly in poorer countries (8). In Bangladesh, consumption of vegetables and fruits and regular exercise are at a low level (24). The use of alcohol is rare but the use of tobacco products, excessive intake of salt, and abuse of substances are substantially high (24). A number of NCDs share these same risk factors, and interventions directed towards these will address these simultaneously (19). Ways of addressing these risk factors need to be innovative and culturally appropriate to the health-promotion initiatives. Prevention, in addition to palliative care, can provide rural people with a better quality of life, reduce unnecessary medical costs and loss of productivity, and strengthen both household and national economies.

The results of the study highlighted the disheartening picture of healthcare-seeking during fatal illness. Although there were some age and sex differences, only 26% of the deceased in 2003-2004 received treatment from physicians before death. Healthcare-seeking patterns have not changed much over the years; 25% of women aged 15-44 years were attended by physicians during fatal illness episodes in Matlab in 1976-1985 (21). Individuals needing medical care consulted local healers or other sources in the majority (60%) of the cases, indicating poor access to the rural health system infrastructure. A specifically-designed study is needed to identify the reasons for such choices and behaviours, as the present study was not aimed at this specific research question.

Particularly in rural areas, the first contact points in the case of sickness are the public and private primary healthcare (PHC) centres that focus mainly on management of infectious diseases that are of utmost concern. The present increasing burden of NCDs in the community demands equipping the PHC centres to manage NCDs in a manner equal to the current management of infectious diseases. Health workers with experience in the prevention and management of chronic NCDs are necessary to impart knowledge of preventive measures to lower risks of NCDs. They can advise patients and caretakers when to adopt palliative care at home to assuage suffering and when to forego care in the home for immediate professional care. As NCDs bring catastrophic economic consequences for households and greatly exacerbate poverty, their control must receive due importance to alleviate poverty and improve population health.

## ACKNOWLEDGEMENTS

This study was funded by ICDDR,B and its donors who provide unrestricted support to the Centre for its operation and research. Current donors providing unrestricted support include: Australian International Development Agency (AusAID), Government of the People's Republic Bangladesh, Canadian International Development Agency (CIDA), Embassy of the Kingdom of the Netherlands (EKN), Swedish International Development Cooperation Agency (Sida), and Department for International Development, UK (DFID). The authors gratefully acknowledge these donors for their support and commitment to the Centre's research efforts, the residents in Matlab HDSS area for continuous cooperation, and HDSS field staff for their hard work.
